# Integrating enzymatic hydrolysis and nanoparticle catalysis for sustainable bioethanol production from pumpkin and dragon fruit pomace by non-conventional yeasts based fermentation prosesses

**DOI:** 10.1007/s11274-026-04818-z

**Published:** 2026-02-12

**Authors:** Aybüke Kut Yılmaz, Ekin Demiray, Sevgi Ertuğrul Karatay

**Affiliations:** 1 Department of Biology, Faculty of Science, Ankara University, Ankara, Beşevler 06100 Turkey; 2 Department of Medical Services and Techniques, Health Services Vocational HighSchool, Ankara Yıldırım Beyazıt University, Ankara, Çubuk 06760 Turkey

**Keywords:** Pumpkin pomace, Dragon fruit pomace, Yeast, Nanoparticles

## Abstract

This study investigated the effects of metal oxide nanoparticles on bioethanol production from lignocellulosic wastes—dragon fruit pomace, and pumpkin pomace—using yeast strains *Saccharomyces cerevisiae*, *Kluyveromyces marxianus*, and *Candida boidinii*. Among the tested substrates, pumpkin pomace yielded the highest ethanol concentrations, particularly at the highest biomass loading (150 g/L). Enzymatic hydrolysis with cellulase was optimized, with 60 FPU/g substrate identified as the most cost-effective loading for maximizing sugar release and ethanol yield. The application of metal oxide nanoparticles (ZnO, Fe_2_O_3_, and NiO) was explored to enhance fermentation efficiency. NiO nanoparticles at 20 mg/100 mL significantly improved bioethanol production. Without supplementation, 21.93 g/L of bioethanol (Y_*P/S max*_: 0.24 g/g, Q_*p max*_: 0.18 g/L.h) was obtained from 150 g/L PP. However, when 20 mg/100 mL NiO nanoparticles were added to a 150 g/L pumpkin pomace medium, and the enzyme loading was adjusted to 60 FPU/g substrate, the ethanol concentration increased by 95.8% to 42.64 g/L. Y_*P/S max*_ and Q_*p max*_ were found to be 0.40 g/g and 0.89 g/L.h, respectively, in these conditions. These results demonstrate that integrating nanoparticle-assisted hydrolysis and fermentation is an effective, cost-saving approach to enhance bioethanol production from low-value agricultural residues, providing a promising approach for sustainable biofuel generation.

## Introduction

The ongoing growth of the global economy has resulted in a phenomenal rise in energy demand, contributing to the accumulation of greenhouse gases. These issues have intensified efforts to develop renewable and sustainable energy alternatives (Medipally et al. 2015). Over the years, renewable energy sources such as wind, geothermal, solar, and biomass have been explored as viable alternatives to fossil fuels. More recently, there has been increasing interest in biomass-based energy generation (Srilatha et al. 2019). Within the spectrum of renewable options, biofuels such as bioethanol, biobutanol, and biodiesel produced from various feedstocks, including agricultural residues, industrial by-products, and municipal waste, have gained significant importance. These bio-based fuels have a lower environmental impact than conventional fossil fuels (Tse et al. 2021).

Bioethanol, one of the most extensively studied biofuels, is regarded as an environmentally sustainable alternative to fossil fuels due to its high oxygen content and relatively low carbon dioxide (CO₂) and carbon monoxide (CO) emissions. These properties significantly reduce the transportation sector’s carbon footprint (Mehani and Bouchekima 2018; Phwan et al. 2018). Bioethanol is classified into four generations based on the type of feedstock used. First-generation bioethanol is produced from food-based crops such as wheat and corn. While first-generation bioethanol offers high conversion efficiency, it is associated with high production costs and concerns regarding food security due to competition between fuel and food supply (Robak and Balcerek 2018). Second-generation bioethanol is derived from non-edible raw materials, including agricultural residues (e.g., straw, molasses), forest by-products, and agro-industrial wastes such as fruit peels and nutshells. Among these, lignocellulosic biomass stands out as the most abundant and promising feedstock for second-generation production. Third-generation bioethanol is produced from photosynthetic microorganisms, particularly cyanobacteria and microalgae, as renewable sources. On the other hand, fourth-generation bioethanol relies on genetically or metabolically engineered crops and microorganisms to enhance yield and process efficiency. Among these various approaches, the use of low-value lignocellulosic waste has gained significant attention due to its availability, cost-effectiveness, and environmental benefits.

Pumpkin is a nutritionally rich vegetable typically grown in tropical and subtropical regions. Its composition allows it to be used for various purposes, including as an antimicrobial, anticancer, antioxidant, and prebiotic agent. In 2024, global pumpkin production was recorded as nearly 27 million tons (Gavril et al. 2024). In the food industry, pumpkin is processed into various forms, such as purée and juice. This processing yields considerable amounts of waste, including seeds, fibrous pomace, and peels. These by-products constitute 18–21% of the total fruit weight, which equates to an annual global generation of 4.86–5.67 million tons of pumpkin waste (Mala and Kurian 2016). In addition to its availability, pumpkin pomace contains approximately 16.42% hemicellulose, 44.23% cellulose, and 19.37% lignin. Given its high cellulose content and availability, it has the potential to serve as a suitable and low-cost feedstock for biofuel production (Atencio et al. 2021; Hussain et al. 2022). In addition to pumpkin, dragon fruit is an interesting option for various applications due to its high vitamin C content and growing global demand. Over the past decade, dragon fruit production has expanded significantly, reaching nearly 4 million metric tons annually. Moreover, fruit processing results in 30–50% of the total dragon fruit weight being converted into lignocellulosic by-products. Given this high waste fraction, the annual potential for dragon fruit pomace is estimated at 1.2–2.0.2.0 million tons. (Thirugnanasambandham et al. 2014; Chen et al. 2024; Du et al. 2024). Because of their abundance, these by-products represent promising raw materials for bioethanol production.

Various methods, including physical, chemical, physico-chemical, and biological pretreatments, are employed during the hydrolysis of lignocellulosic biomass. Among these, the most commonly used approach involves the combined hydrolysis of lignocellulose with dilute acids and enzymes, such as cellulases. This method is highly effective at releasing fermentable sugars during fermentation, as it efficiently disrupts the lignin, hemicellulose, and cellulose structures. Due to its relative cost-effectiveness and high efficiency, the combined use of dilute sulfuric acid and enzymatic hydrolysis has been widely applied not only in scientific research but also in industrial-scale bioethanol production (Aden and Foust 2009).

Nanoparticles are promising materials for bioethanol production because of their high surface area-to-volume ratio and unique physicochemical properties, enabling their widespread application in fields such as medicine, energy, electronics, environmental remediation, and biotechnology (Khan et al. 2019). Due to their small size and high reactivity, nanoparticles have also been reported to enhance the overall efficiency of bioethanol production by improving enzymatic hydrolysis and the fermentation process (Verma et al. 2022). Nanoparticles can significantly enhance the enzymatic hydrolysis efficiency of lignocellulosic biomass through several mechanisms. For instance, these molecules provide a large surface area for immobilizing enzymes, helping maintain their structural integrity and catalytic activity. They can also enhance the interaction between cellulolytic enzymes and lignocellulosic biomass by reducing mass-transfer limitations and preventing nonproductive binding between enzymes and lignin. In this way, nanoparticles may help disrupt the compact structure of cellulose via increasing surface accessibility for enzymes (Ingle et al. 2018; Bidir et al. 2021; Kotwal et al. 2024). These substances not only improve enzymatic hydrolysis but also fermentation by affecting both biological and process-related parameters. Nanoparticles increase surface area and enhance substrate-biomass contact, facilitating faster, more efficient nutrient uptake. They also immobilize enzymes or microbes on nanoparticles to stabilize them and prolong their activity. Moreover, some nanoparticles can act as mild stressors, triggering adaptive metabolic responses and increasing metabolite production (Aamer et al. 2025).

For the reasons mentioned, the current study investigated the production of bioethanol from dragon fruit pomace (DFP) and pumpkin pomace (PP) using the yeast strains *S. cerevisiae*,* K. marxianus*, and *C. boidinii*. To enhance the overall efficiency of enzymatic hydrolysis and fermentation, selected nanoparticles (Fe2O3, NiO, and ZnO) were incorporated into both the raw material hydrolysis and the fermentation process. To the best of our knowledge, this is the first report about the effect of metallic nanoparticles (Fe2O3, NiO, and ZnO) on the enzymatic hydrolysis of PP.

## Materials and methods

### Microorganisms and raw materials

The yeast strains *S. cerevisiae*, *K. marxianus*, and *C. boidinii* were obtained from the Ankara University Culture Collection. The yeast cultures were maintained on potato dextrose agar (PDA) medium at 4 °C until use.

Dragon fruit pomace (DFP) was obtained from a local supplier in İzmir/Turkey. Pumpkin pomace (PP) was obtained from a local company in Ankara/Turkey. All raw materials were dried overnight in an oven at 70 °C and ground in a laboratory-type mill (Miprolab, 0.2 cm mesh size).

### Pretreatment of raw materials

#### Physicochemical pretreatment

In the first part of the experiments, liquid fractions were used in order to determine the reducing sugar content of the raw materials. For this purpose, increasing concentrations (50, 100, 150 g/L) of DFP and PP were pretreated with 1% H₂SO₄ (w/v) and applied in an autoclave at 121 °C for 15 min based on the solids content (ALP/CL-40 M, Germany). Following the pretreatment, all raw materials were filtered at ambient temperature using Whatman No. 1 filter paper, and the resulting liquid fractions were adjusted to pH 5, sterilized in an autoclave at 121 °C for 15 min, and then used for fermentation.

#### Enzymatic hydrolysis

PP was hydrolyzed using Cellic CTec2 (Sigma, 144 FPU/mL), a commercial cellulase. To determine the highest fermentable sugar content at the optimum PP loading, four enzyme concentrations (15, 30, 60, and 120 FPU/g substrate) were tested using the whole biomass fraction of PP.

During enzymatic hydrolysis, the medium pH was adjusted to 4.8 with 50 mM citrate buffer. Enzymatic hydrolysis was performed at 50 °C for 72 h (Adney and Baker 2008). To stop the enzymatic reaction, the hydrolysates were boiled in a water bath for 10 min. The enzyme concentration that resulted in the highest bioethanol production was used for further experiments. To investigate the effect of nanoparticles on enzymatic hydrolysis and pretreatment processes, 3 different concentrations (10, 20, and 40 mg/100 mL) of Fe_2_O_3_, NiO, and ZnO metal nanoparticles were added to the enzymatic hydrolysis environment.

### Fermentation conditions

Yeast cells were precultured in YPD medium for 24 h at 30 °C and subsequently inoculated into the fermentation medium at 10% (v/v). The media pH was set to 4.8. Fermentation experiments were carried out for 72 h. Agitation speed was 150 rpm.

### The effect of initial biomass loading on bioethanol production

To evaluate the effect of initial biomass loading on bioethanol production, three different initial biomass loadings were tested for each raw material: 50 g/L, 100 g/L, and 150 g/L. All experiments were carried out in 100 mL Erlenmeyer flasks with a working volume of 40 mL. Only liquid fractions were used during the experiments.

### Effect of supplements and metal nanoparticles on bioethanol production during fermentation

To compare the effect of conventional supplements and nanoparticles, four different media were prepared as follows: Medium 1: Only pretreated PP with increased biomass loadings (50–150 g/L), Medium 2: 150 g/L enzymatically hydrolyzed PP (15–120 FPU/g substrate) without any supplementation, Medium 3: 150 g/L enzymatically hydrolyzed PP (60 FPU/g substrate) and 5 g/L peptone, 3 g/L yeast extract, 0.5 g/L MgSO₄·7 H₂O, 1 g/L KH₂PO₄, 0.1 g/L CaCl₂, and 0.05 g/L ZnSO₄ and Medium 4: 150 g/L enzymatically hydrolyzed PP (60 FPU/g substrate) contained only Fe_2_O_3_, only NiO or only ZnO nanoparticles). All experiments were performed in triplicate.

*K. marxianus* previously cultivated in YPD medium for 24 h was inoculated into the fermentation medium at a rate of 10% (v/v). Samples were collected at 12, 24, 48, and 72-hour intervals to monitor microbial growth, sugar consumption, and ethanol production. Fermentation experiments were carried out at 30 °C agitation at 100 rpm.

### Analytical methods

Ethanol concentrations were determined using gas chromatography (GC-2010, Shimadzu). Prior to GC analysis, 1.5 mL of each sample was centrifuged at 10,000 rpm for 10 min (Hettich, Germany). Bioethanol was determined with GC (Shimadzu, Japan). The supernatant was filtered through a 0.22 μm membrane filter, and 1 µL of the filtrate was injected into the injection unit (SPL). A Restek Rtx-Wax capillary column (60 m length, 0.25 mm i.d.) and a flame ionization detector (FID) were used for ethanol analysis. The injection port and detector temperatures were set to 140 °C and 160 °C, respectively. The initial column temperature was 50 °C, which was increased to 150 °C over 19 min. The column flow rate was 1.86 mL/min, and nitrogen gas was used as the carrier gas (Wistara et al. 2016).

Sugar profiling of the raw materials was performed with a high-performance liquid chromatography (HPLC) system (Shimadzu/Japan) equipped with a Coregel 87H3 column (Transgenomic) and a refractive index detector (RID). Before analysis, 1.5 mL of each sample was centrifuged at 10,000 rpm for 10 min. The supernatant was filtered through a 0.22 μm membrane filter. The column oven temperature was maintained at 80 °C, with a total flow rate of 0.5 mL/min over 30 min. A 5 mM H₂SO₄ solution was used as the mobile phase. (Motoda et al. 2019).

Microbial growth was measured by examining the samples’ optical density (OD) at 600 nm using a spectrophotometer (Shimadzu/Japan). The results obtained from the spectrophotometer were converted to g/L using the microorganism standard curve generated subsequently.

Total reducing sugars were determined using the DNS (3,5-dinitrosalicylic acid) method (Miller 1959). The filter paper unit (FPU) of the enzyme was measured according to the method described by Adney and Baker (2008). Theoretical ethanol yields were calculated using Eq. 1 (Kim and Lee 2007):

1 $$\begin{array}{l}Percentage\:of\:theoretical\:ethanol\:yield\left(\%\right)\\=\frac{\mathrm{ethanol}\:(\mathrm g/\mathrm L)\:}{\mathrm{initial}\:\mathrm{sugar}\:(\mathrm g/\mathrm L)\:\:\times\:0.511\:}\times\:100\end{array}$$

Volumetric ethanol productivity (Qp) was calculated according to Eq. 2 (Roca and Olsson 2003):

2 $$\:Q_p\:(\frac{\mathrm{g}}{\mathrm{L}}.\mathrm{h})=\frac{\mathrm{e}\mathrm{t}\mathrm{h}\mathrm{a}\mathrm{n}\mathrm{o}\mathrm{l}\:(\mathrm{g}/\mathrm{L})\:}{t_{maximum\:ethanol}\:}$$

Ethanol yields (Y_*P/S*_) were determined using Eq. 3 (Yücel and Aksu 2015).

3 $$\:{Y}_{P/S}\:(\mathrm{g}/\mathrm{g})=\frac{\:\mathrm{e}\mathrm{t}\mathrm{h}\mathrm{a}\mathrm{n}\mathrm{o}\mathrm{l}\:(\mathrm{g}/\mathrm{L})\:}{\mathrm{c}\mathrm{o}\mathrm{n}\mathrm{s}\mathrm{u}\mathrm{m}\mathrm{e}\mathrm{d}\:\mathrm{s}\mathrm{u}\mathrm{g}\mathrm{a}\mathrm{r}\:(\mathrm{g}/\mathrm{L})\:}$$

Cellulose concentration of raw pumpkin pomace was determined according to the standard ISO protocol (ISO 5498:1981). Cellulose determination was performed by an external laboratory, namely Düzen Norwest, Ankara.

### Statistical analyses

Prior to conducting ANOVA, the assumptions of normal distribution of the data and homogeneity of variances were evaluated using the Shapiro–Wilk and Levene tests, respectively. Since all datasets satisfied the assumptions for parametric testing, data were assessed using one-way analysis of variance (ANOVA). When significant effects were detected (*p* < 0.05), Tukey’s honestly significant difference (HSD) test was applied for post-hoc pairwise comparisons. Results are presented as mean ± standard deviation, and statistically different groups in the same column were labeled using superscript letters, where identical letters denote no significant difference and different letters indicate significant differences among treatments. All statistical analyses were performed using R (version 4.5.1).

## Results and discussion

### Comparison of fermentable sugar and bioethanol production of raw materials by different yeasts

**Fig. 1:**
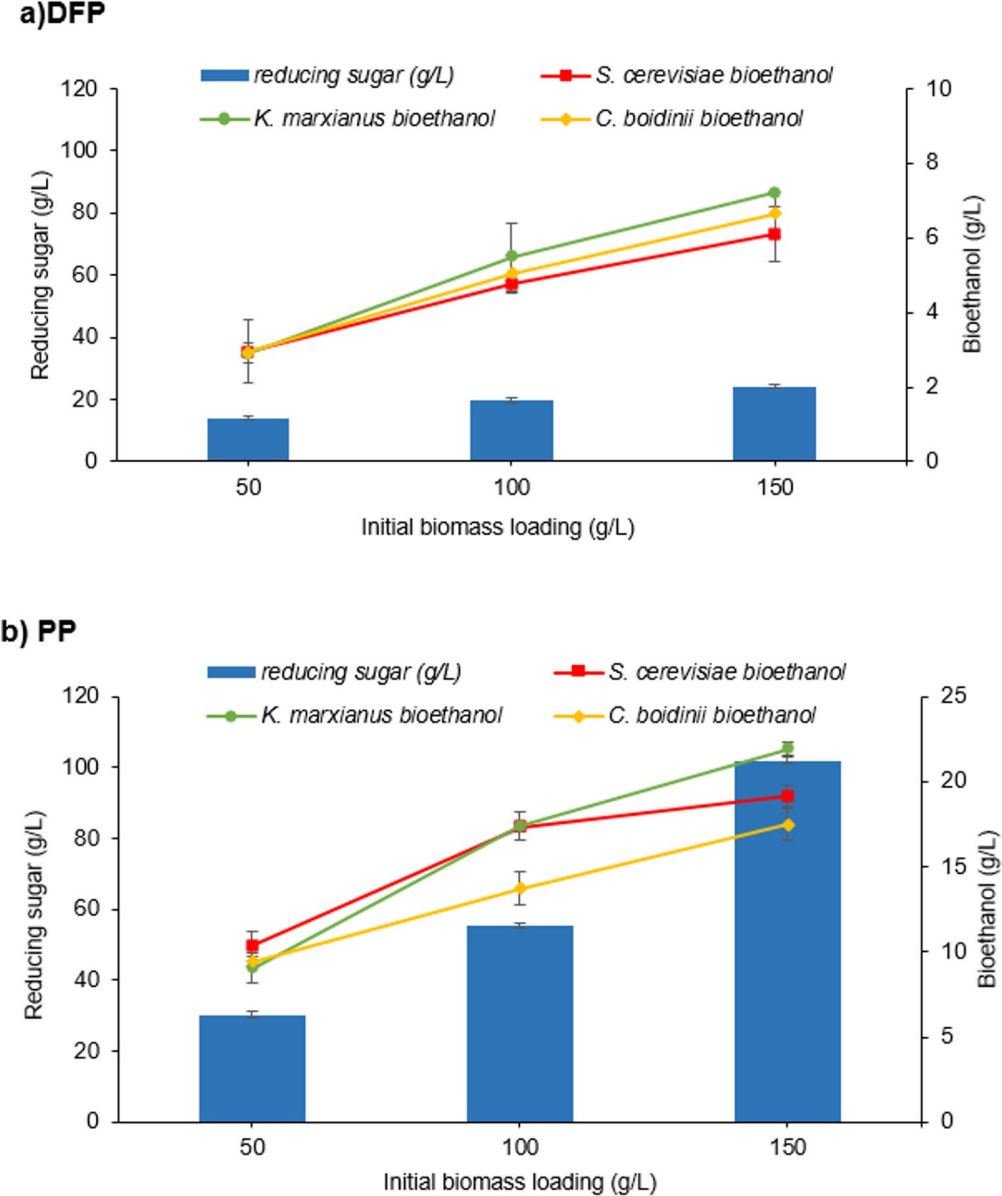
Bioethanol production by *S. cerevisiae*,* K. marxianus*, and *Candida boidinii* at initial biomass loadings of dragon fruit pomace and pumpkin pomace (**a)**: Dragon Fruit Pomace (**b)**: Pumpkin Pomace (Pretreatment conditions: 1% H_2_SO_4_, 121 °C, 15 min, pH:5,fermentation time: 24 h for *K. marxianus*, *S. cerevisiae* and *C. boidinii*)

In this part of the study, increasing concentrations (50, 100, 150 g/L) of two different biomass types (PP and DFP) were tested for fermentable sugar concentrations and bioethanol production using *S. cerevisiae*, *K. marxianus*, and *C. boidinii*. Figure 1 presents a comprehensive comparison of bioethanol production and reducing sugar levels for DFP and PP, respectively.

The experiments were conducted using three different initial biomass loadings: 50 g/L, 100 g/L, and 150 g/L. This range of biomass loadings allows for a thorough examination of how substrate concentration affects ethanol production and sugar utilization by the yeast strains. A clear trend emerges across all raw materials: as the initial biomass loading increases from 50 g/L to 150 g/L, ethanol production and reducing sugar levels both increase. This positive correlation suggests that higher substrate availability is associated with enhanced fermentation performance and increased sugar release from lignocellulosic biomass. Furthermore, the lowest reducing sugar concentrations were observed as 13.91 g/L (0.278 g/g _biomass_), and 30.23 g/L (0.604 g/g _biomass_) for DFP and, PP at 50 g/L initial biomass loadings and these values reached their maximum when initial biomass loading increased to 150 g/L as 24.24 g/L (0.161 g/g _biomass_), and 101.93 g/L (0.679 g/g _biomass_), respectively. These data demonstrate that the change in the substrate-to-product yield (g/gbiomass) with increased biomass loading shows material-specific efficiency, increasing for PP but decreasing for DFP, suggesting potential substrate inhibition or mass transfer limitations in the latter.

Similarly, the lowest ethanol production was obtained with 50 g/L biomass loading across all raw materials.In the presence of 50 g/L DFP, the lowest bioethanol yield was observed as 2.90 g/L (0.058 g/g _biomass_) by *K. marxianus*. In contrast, *S. cerevisiae* yielded higher ethanol, such as 9.05 g/L (0.058 g/g _biomass_) from 50 g/L PP. Ethanol production by *C. boidinii* was determined as 2.94 (0.058 g/g _biomass_) g/L and 9.43 g/L (0.188 g/g _biomass_) in 50 g/L DFP and PP, respectively. Conversely, when 150 g/L biomass was used, ethanol production increased dramatically, with the highest ethanol titers of 7.20 g/L (0.048 g/g biomass) and 21.93 g/L (0.146 g/g biomass) from *K. marxianus* for the respective raw materials.The ethanol yield (g/g _biomass_) showed a detrimental correlation with increasing biomass loading from 50 g/L to 150 g/L for both raw materials and yeasts tested, as evidenced by the marked decrease in g/g performance, despite the simultaneous rise in ethanol concentration (g/L); this efficiency drop is likely attributable to factors associated with high-gravity fermentation, and dfmuemonstrates that the yeasts are beginning to be constrained (or inhibited) in its bioethanol production performance. Among the raw materials, PP demonstrates the most promising results for ethanol production. At the highest biomass loading of 150 g/L, PP yields ethanol concentrations of 21.93 g/L (0.146 g/g _biomass_). This superior performance may be attributed to the specific composition of PP, which could provide a more favorable environment for yeast fermentation or contain higher levels of fermentable sugars (Genevois et al. 2016; Wang et al. 2023). The trend toward reduced sugar levels with higher biomass loadings was also observed for DFP. This phenomenon indicates that more fermentable sugars become available as the biomass concentration increases. The release of these sugars could be due to improved mass transfer at higher solid loadings, providing the yeast with more accessible carbon sources for fermentation (Gong et al. 2023). At this stage of the study, *K. marxianus* produced the highest ethanol concentration of 21.93 g/L with a biomass loading of 150 g/L PP, whereas it yielded only 7.2 g/L ethanol at the same DFP concentration. Furthermore, across all tested concentrations, higher ethanol titers were observed in PP; thus, *K. marxianus* and PP biomass were selected for subsequent experiments.

The data presented in this section provide valuable insights for optimizing bioethanol production from DFP and PP as raw materials. The results demonstrate that higher initial biomass loadings lead to increased ethanol yields, suggesting that more concentrated fermentation processes are more efficient. However, it’s important to note that there may be practical limitations to increasing biomass loading indefinitely, such as mass transfer limitations or increased viscosity of the fermentation broth. Therefore, biomass concentrations above 150 g/L were not tested.

The experimental results confirm that both raw materials are suitable for bioethanol production. However, given its superior global market position, pumpkin pomace (PP) has greater potential than dragon fruit pomace (DFP). The estimated waste ratio of approximately 18–21% generated from the global production of nearly 27 million tons of pumpkin provides excellent economic scale for biorefineries. Furthermore, PP’s composition is highly favorable for conversion, possessing a high cellulose content and a relatively low lignin content, which facilitates the use of lower-cost, conventional pretreatment strategies to release fermentable sugars. Consequently, this by-product is frequently utilized for bioethanol production in the literature (Chouaibi et al. 2020; Bashir et al. 2025). In contrast, DFP’s annual production is lower than PP’s. While this high conversion ratio and concentration in key Asian processing hubs translate to lower collection and transportation costs for localized facilities, the pomace’s structural complexity as a thick peel, rich in pectin and pigments, may necessitate more intensive and potentially costlier pretreatment to achieve optimal ethanol yields. In summary, PP is better suited to large-scale, global production due to its high volume and favorable composition, making it the optimal feedstock for further study.Given their zero cost and the environmental issues associated with them, valorizing these wastes for renewable energy production is particularly important. In this context, waste disposal was achieved using a biorefinery approach in this study.

### Effect of enzyme loading on sugar release, ethanol Yield, and microbial growth

**Fig. 2:**
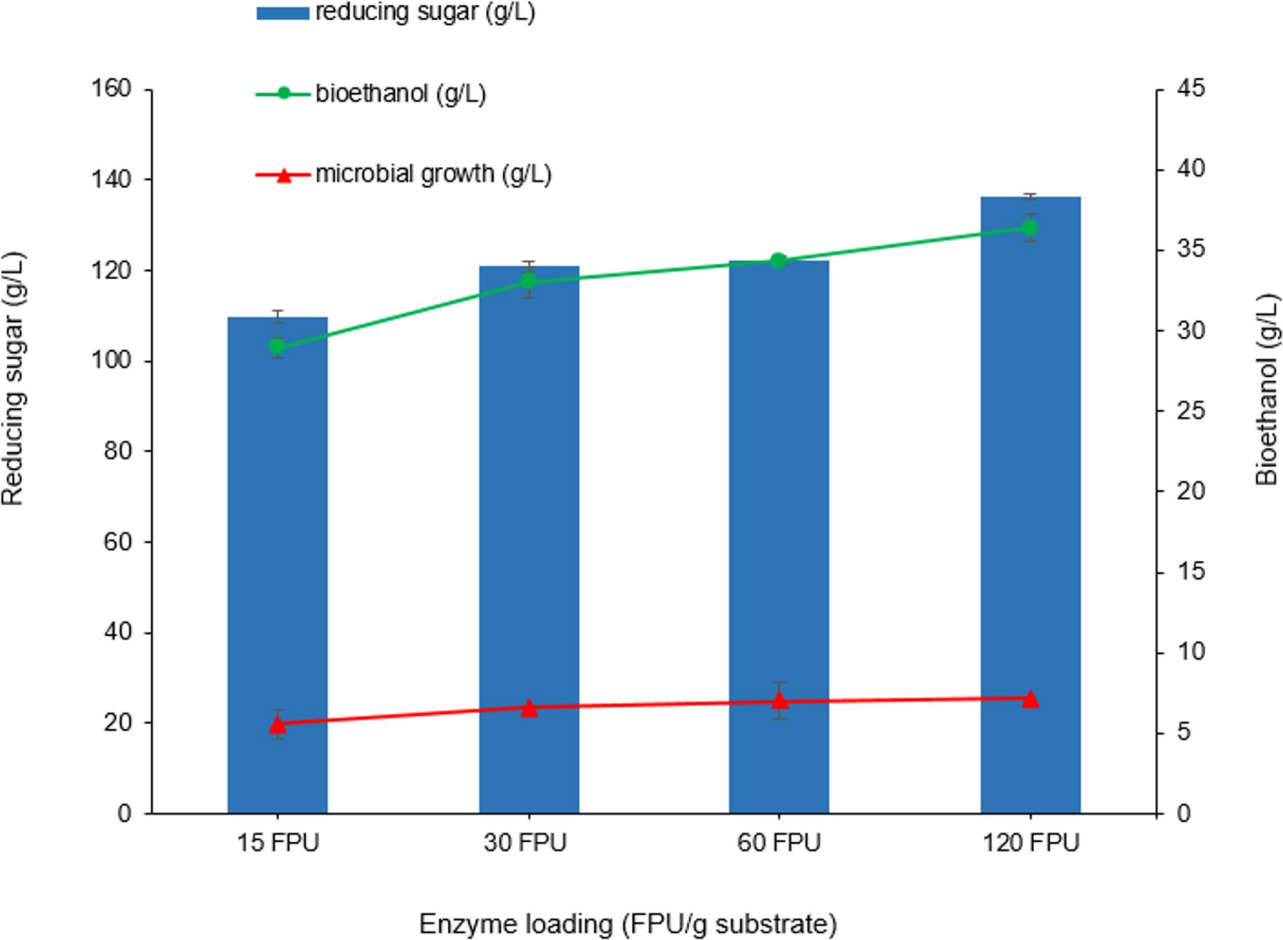
Effect of enzyme loadings on sugar concentration of pumpkin pomace and bioethanol production of *K. marxianus* (Pretreatment conditions: 1% H_2_SO_4_, 121 °C, 15 min, biomass loading:150 g/L, fermentation time: 48 h for *K. marxianus*, 48 h)

The influence of increasing enzyme loading (15–120 FPU/g substrate) on the efficiency of enzymatic hydrolysis and downstream fermentation was evaluated using 150 g/L PP as the substrate (Medium 2). The resulting reducing sugar concentration, ethanol production, and microbial growth were quantified to assess overall process performance (Fig. 2). As expected, reducing sugar concentration increased steadily with increasing enzyme loading. At 15 FPU/g substrate, hydrolysis yielded 109.73 g/L reducing sugars, which rose to 145.57 g/L at 120 FPU/g substrate. This trend confirms that higher enzyme doses enhance polysaccharide degradation, improving initial fermentable sugar availability, and demonstrates the critical role of enzyme loading in maximizing hydrolysis performance. Similarly, in a previous study, enzymatic hydrolysis of brewer’s spent grain cellulose showed a marked increase in glucose yield and cellulose conversion with higher enzyme loadings. Similarly, in a study conducted by Mussatto et al. (2008), enzymatic hydrolysis of brewer’s spent grain cellulose showed a marked increase in glucose yield and cellulose conversion with higher enzyme loadings. For example, at an enzyme concentration of 45 FPU/g, glucose yield reached 93.1%, compared to significantly lower values at 5 FPU/g. Conversely, increasing the substrate concentration from 2% to 8% (w/v) decreased hydrolysis efficiency, likely due to increased viscosity and mass-transfer limitations.

In parallel, ethanol production also exhibited a positive correlation with enzyme concentration, rising from 28.94 g/L at the lowest enzyme dose to 42.61 g/L at 120 FPU/g substrate. This increase indicates that enhanced saccharification directly benefits fermentation, likely because higher sugar concentrations facilitate sustained yeast activity and ethanol synthesis.

Interestingly, microbial biomass formation showed only a modest increase across enzyme loading levels, reaching a peak at 30–60 FPU/g substrate. In the presence of 15 FPU/g substrate, microbial growth of K. marxianus was 5.6 g/L, and this value increased to 7.14 g/L when the enzyme concentration increased to 120 FPU/g substrate. This suggests that yeast growth may not depend solely on sugar availability, and factors such as nutrient limitation, ethanol toxicity, or oxygen transfer may have constrained further biomass accumulation at higher enzyme dosages. Collectively, these findings demonstrate that increasing cellulase dosage improves both sugar release and ethanol yield, with diminishing returns on microbial growth. While 120 FPU/mL achieved the highest ethanol output, the moderate gains compared to 60 FPU/mL suggest that process optimization should balance enzyme cost with output efficiency. In a study by Littlewood et al. (2013), hot-water pretreatment at 190 °C for 10 min increased total sugar yield from bamboo, and this condition was identified as the optimal pretreatment. Under these conditions, the bioethanol production cost was approximately 0.484 $/L at the optimal enzyme loading (60 FPU/g glucan), demonstrating that bioethanol could become competitive with gasoline. Furthermore, it was reported that the highest enzyme loading at which bioethanol could remain cost-competitive with gasoline was 60 FPU/g glucan, and that production costs remained feasible up to this level.

The results demonstrate that while moderate enzyme levels (60 FPU/g substrate) achieved high sugar-to-ethanol conversion efficiency (highest Y_*P/S*_), higher enzyme concentrations (120 FPU/g substrate) maximized volumetric productivity and theoretical yield. Thus, the choice of enzyme loading should balance costs, conversion efficiency, and productivity targets depending on the economic and operational priorities of the process. For the aforementioned reasons, 60 FPU/g substrate was selected for further studies.

### Comparative evaluation of metal oxide nanoparticles on ethanol fermentation efficiency

**Fig. 3:**
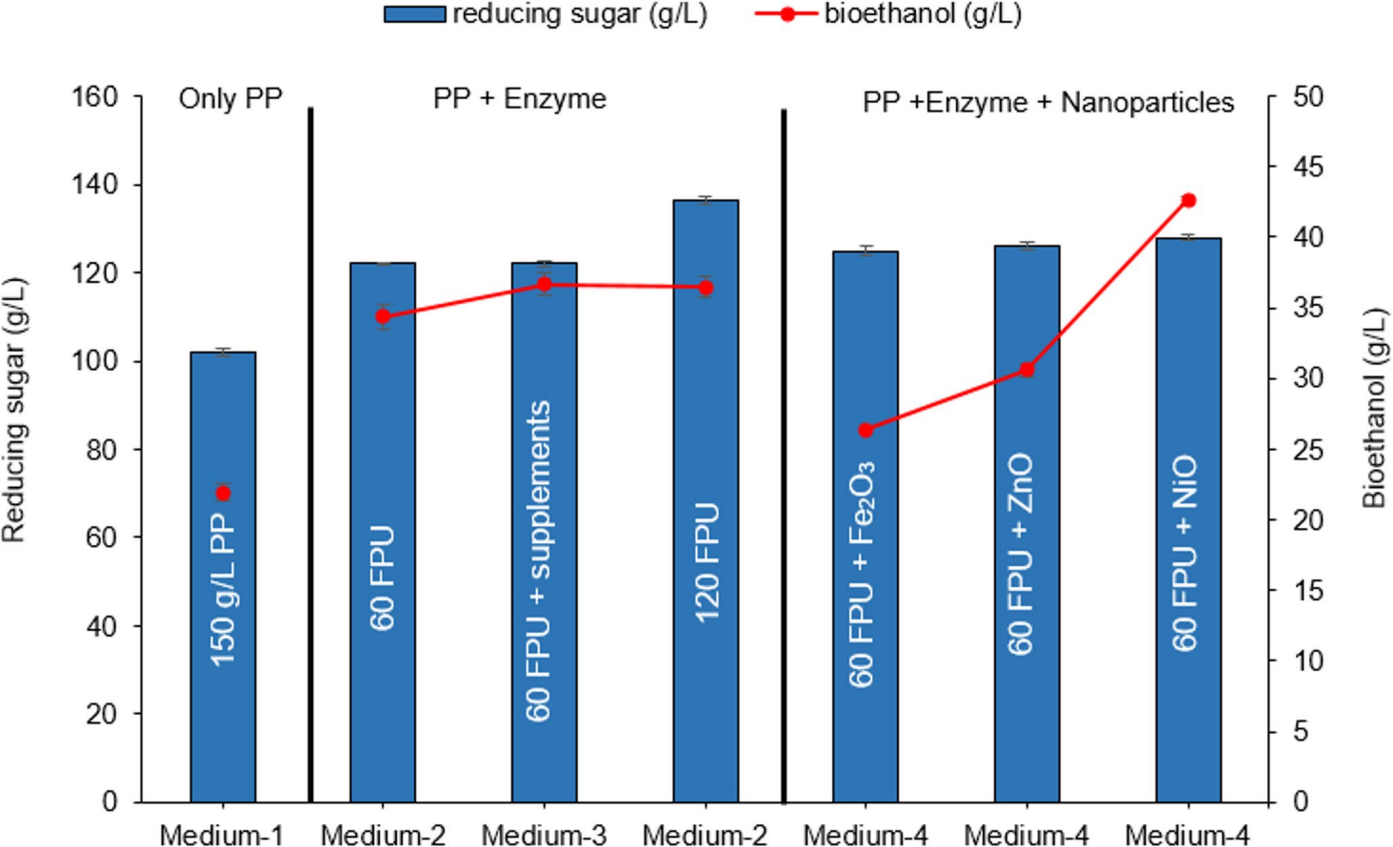
Effect of enzyme loadings and nanoparticle on sugar concentration of pumpkin pomace and bioethanol productions of *K. marxianus* (**Medium 1**: Only pretreated PP, **Medium-2**: Enzymatically Hydrolyzed PP without any supplementation, **Medium-3**: Enzymatically hydrolyzed PP (60 FPU/g substrate) and 5 g/L peptone, 3 g/L yeast extract, 0.5 g/L MgSO₄·7 H₂O, 1 g/L KH₂PO₄, 0.1 g/L CaCl₂, and 0.05 g/L ZnSO₄ and **Medium-4**: Enzymatically hydrolyzed PP (60 FPU/g substrate) and 20 mg/100 mL nanoparticles)

In this part of the experiments, the effects of nanoparticles on fermentation efficiency were compared with conventional media types since these nanoparticles are stable against denaturation, appropriate for application in the presence of high biomass concentrations, cost-effective, and easy to process (Fan et al. 2014; Ovais et al. 2018; Chen et al. 2017). For this purpose, four different media were used to compare the effect of NiO nanoparticles with that of conventional media. In this context, Fig. 3 compares reducing sugar concentrations and bioethanol yields across various fermentation conditions using different enzyme dosages and the presence or absence of metal oxide nanoparticles. Medium 1, which contained only 150 g\L PP without enzyme treatment, yielded 101.93 g\L reducing sugar and 21.93 g/L bioethanol. Furthermore, Medium 2 (only enzymatically hydrolyzed PP with 120 FPU/substrate cellulase) yielded the highest reducing sugar concentration, as 136.26 g/L. Medium 2 and Medium 3 (containing enzymatically hydrolyzed PP and supplements), hydrolyzed with 60 FPU/g substrate, produced lower ethanol levels (36.69 g/L and 34.36 g/L), respectively. Conversely, the maximum ethanol production (42.64 g/L) was observed in Medium-4 (60 FPU/g substrate/20 mg/100 mL NiO-containing group), suggesting that nanoparticle addition and medium composition both influence fermentative efficiency.

In the nanoparticle-assisted conditions (Medium 4), the enzyme loading was kept constant at 60 FPU/mL. The addition of NiO, ZnO, and Fe_2_O_3_ nanoparticles maintained relatively high reducing sugar concentrations (127.82 g/L, 123.30 g/L and 114.95 g/L), but bioethanol production declined in the presence of ZnO and Fe_2_O_3_, respectively. The presence of NiO nanoparticles resulted in the highest ethanol titer among the nanoparticle-treated samples (42.64 g/L). In contrast, ZnO and Fe_2_O_3_ yielded significantly lower ethanol concentrations (30.58 g/L and 26.39 g/L, respectively), despite the similar sugar presence. These results suggest that while metal oxide nanoparticles do not hinder enzymatic saccharification, their effect on ethanol production is nanoparticle-specific. NiO nanoparticles may enhance or at least preserve fermentation efficiency, potentially by mitigating inhibitors or stabilizing enzymatic activity. Conversely, ZnO and Fe_2_O_3_ may inhibit microbial metabolism or fermentation pathways. The superior performance observed with NiO may be attributed to improved enzyme–substrate interactions, which led to better disruption of the biomass structure. NiO might also create a more favorable microenvironment, thereby promoting the catalytic activity of cellulolytic enzymes and the uptake of fermentable sugars by yeast cells. Similar positive effects of NiO nanoparticles on sugar and ethanol yields were also previously reported by Sanusi et al. (2019), who showed that NiO nanoparticles at 0.01 wt% enhanced ethanol production by increasing yeast growth. These results show that NiO is the most promising additive among the tested metal oxide nanoparticles for increasing ethanol production under enzyme-limited conditions, making it a potential candidate for cost-effective, high-yielding bioethanol processes. Therefore, the effect of varying NiO concentrations on *K. marxianus* fermentation was examined in subsequent steps.

### Effect of nanoparticle concentrations on ethanol production

**Fig. 4:**
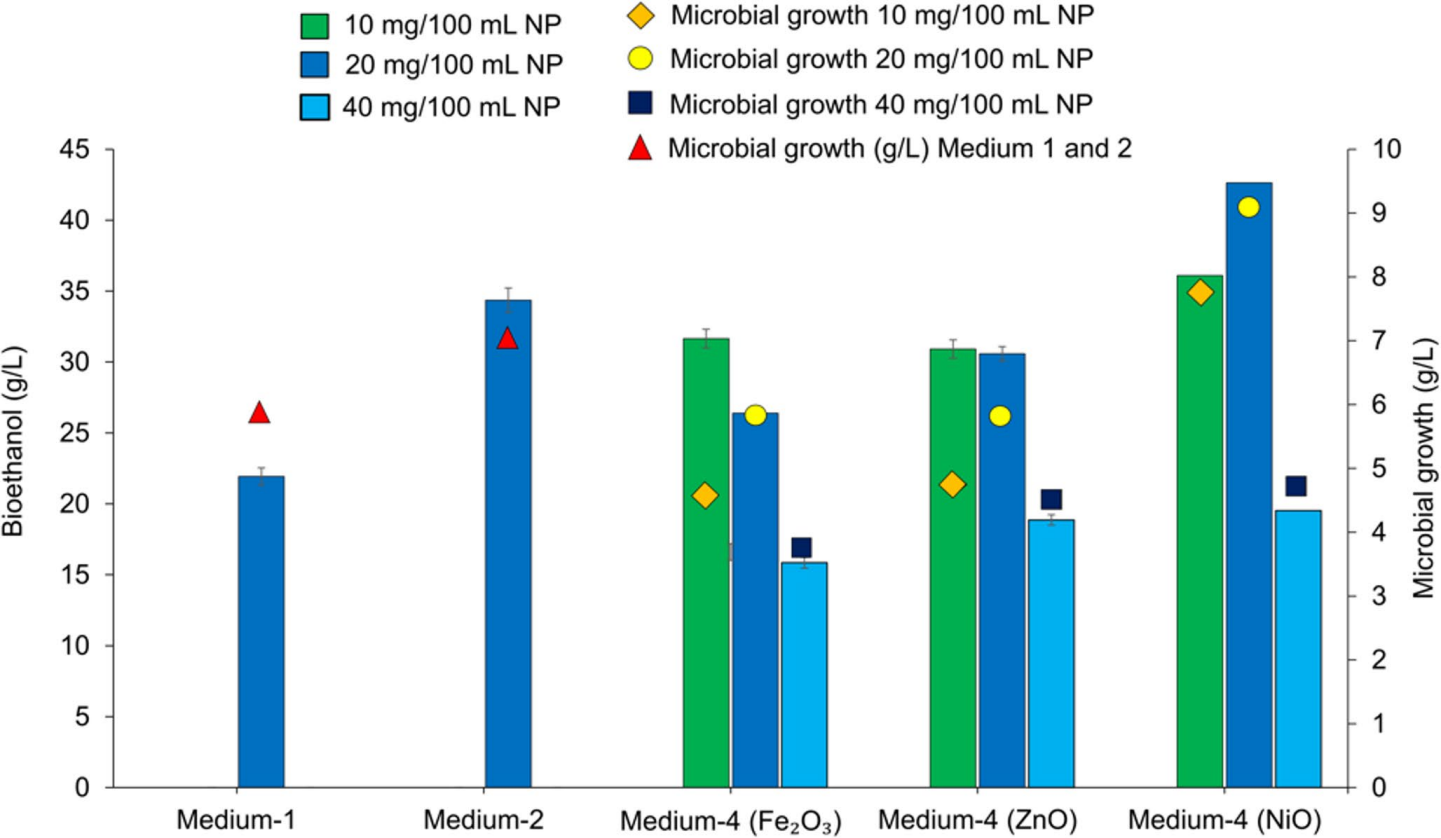
Effect of different nanoparticles (NiO, ZnO and Fe_2_O_3_) on sugar concentration of pumpkin pomace and bioethanol productions of *K. marxianus* (Pretreatment conditions: 1% H_2_SO_4_, 121 °C, 15 min, biomass loading:150 g/L, enzyme concentration: 60 FPU pH:4.8, fermentation time: 48 h for *K. marxianus* **Medium 1**: Only pretreated PP, **Medium-2**: Enzymatically Hydrolyzed PP without any supplementation, **Medium-4**: Enzymatically hydrolyzed PP (60 FPU/g substrate) and 20 mg/100 mL nanoparticles)

The effects of varying concentrations of metal oxide nanoparticles (10–40 mg/100mL) on ethanol production were investigated. The results, presented in Fig. 4, demonstrated a strong dependence of ethanol yield on both nanoparticle dosage and fermentation time, with the 20 mg/100 mL NiO treatment achieving the highest ethanol concentration of 42.64 g/L at 48 h—significantly surpassing other conditions. This suggests that 20 mg/100 mL is an optimal nanoparticle concentration that enhances fermentation kinetics, potentially by facilitating improved enzyme–substrate interactions. Supporting these findings, a previous study reported that cellulase enzymes immobilized on graphene oxide coated with nickel-based nanoparticles (NiFe₂O₄ and Fe₃O₄) exhibited enhanced stability and catalytic efficiency during the hydrolysis of rice straw. In parallel with previous observations in the literature, current findings indicate that nickel-containing nanoparticles can positively influence enzymatic hydrolysis and fermentation processes in lignocellulosic biomass conversion. This positive effect can be explained by several factors. Firstly, the high surface area of NiO can promote better cellulase adsorption and partial disruption of cellulose structure, increasing enzyme accessibility. Secondly, NiO surfaces can also stabilize enzymes through micro-pH buffering and mild redox interactions, thereby increasing hydrolytic efficiency. Although an appropriate NiO concentration does not directly affect fermentation, the increased release of fermentable sugars and limited adsorption of inhibitors can improve ethanol yield (Sanusi et al. 2020; Nduka et al. 2024; Nayeri et al. 2023). At 48 h of fermentation time, the maximum ethanol concentration was 42.64 g/L in the presence of 20 mg/100 mL NiO. This value is higher than that observed with the 10 mg/100 mL (36.09 g/L) and 40 mg/100 mL (19.53 g/L) NiO groups, respectively. Notably, the use of the 40 mg/100 mL nanoparticle suggests a potential inhibitory effect at higher nanoparticle concentrations, possibly due to metal ion toxicity or nanoparticle aggregation interfering with fermentation. Previous studies have reported similar findings, showing that NiO concentrations above 0.2% (wt) inhibit bioethanol production by *Pichia kudriavzevii* IFM 53,048. This decrease in production is attributed to the toxic effects of elevated NiO nanoparticle levels on yeast metabolism and cell viability (Nduka et al. 2024). The positive role of NiO nanoparticles in fermentation was previously reported by Sanusi et al. (2019), who investigated the effects of nine metal oxide nanoparticles (NPs) on ethanol production by *S. cerevisiae BY4743*. The same study demonstrated that when NiO NPs were included as catalysts during the pretreatment of 100 g/L potato waste, the optimum bioethanol concentration was 36.04 g/L. In another study, NiO nanoparticles, particularly at a low concentration (15 µg/µL), enhanced the enzymatic hydrolysis of elephant grass by increasing cellulase activity through improved substrate surface accessibility and the adsorption of lignin/hemicellulose-derived inhibitors, thereby reducing enzyme inhibition. As a result, sugar liberation was maximized at the mentioned NP concentration (Iyyappan et al. 2023).

In contrast to NiO, ZnO, and Fe₂O₃ nanoparticles, which exhibited comparatively lower stimulatory effects on bioethanol production. For ZnO, bioethanol concentrations at 20 and 40 mg/100 mL were 30.58 and 18.87 g/L, respectively, with the highest yield of 30.91 g/L observed at 10 mg/100 mL. In the literature, previous reports indicate that ZnO nanoparticles at concentrations greater than 10 mg/100 mL exhibit an inhibitory effect on bioethanol production. This trend suggests that Zn²⁺ ions released from ZnO nanoparticles may have interfered with yeast metabolism or enzyme function during fermentation (Zada et al. 2014). Similar to ZnO, in the case of Fe₂O₃, a decreasing trend was evident with increasing nanoparticle loading: the maximum bioethanol concentration (31.65 g/L) was obtained at 10 mg/100 mL, whereas increasing the concentration to 40 mg/100 mL reduced the yield to 15.85 g/L. This decline with higher Fe₂O₃ dosage may be attributed to non-selective sugar adsorption or restricted enzyme accessibility caused by particle overloading, as excessive iron nanoparticle usage has previously been shown to lead to sugar loss during enzymatic hydrolysis (Adebule et al. 2024). Similar to ethanol concentrations, microbial growth of *K. marxianus* was influenced by increased NP concentrations. For instance, in 10 mg/100 mL Fe_2_O_3_, ZnO and NiO, the dry weight of the cells was determined to be 5.72 g/L, 5.79 g/L and 7.79 g/L, respectively. These values decreased to 3.69 g/L, 4.32 g/L, and 4.80 g/L when the same NP concentrations were increased to 40 mg/100 mL. On the other hand, the use of 20 mg/100 mL NiO led to the formation of the maximum dry weight, which was recorded at 8.90 g/L, whereas microbial growth in Fe_2_O_3_ and ZnO was observed at 4.60 g/L and 4.62 g/L, respectively.

This improvement highlights the superior compatibility of NiO nanoparticles with the enzymatic hydrolysis–fermentation system under the studied conditions. Overall, the data indicate that the type and concentration of nanoparticles play a critical role in modulating bioethanol productivity, with NiO at 20 mg/100 mL emerging as the optimal condition in this experimental setup. In summary, 20 mg/100 mL NiO yielded the highest ethanol production, highlighting the beneficial effect of moderate nanoparticle supplementation. However, exceeding this optimal concentration (e.g., 40 mg/100 mL) resulted in diminished performance, likely due to adverse effects on microbial growth or fermentation performance. These findings emphasize the importance of dose optimization when applying metal oxide nanoparticles in biotechnological processes.

### Effect of NiO nanoparticles on fermentation inhibitors and reducing sugar content in PP hydrolysates

The effect of increasing concentrations of NiO nanoparticles on the inhibitor levels in pumpkin pomace hydrolysates (150 g/L) was evaluated for HMF, formic acid, and acetic acid (Table 1). The data demonstrated that NiO supplementation influences the formation of fermentation inhibitors. In the untreated control (0 mg/100 mL NiO), the hydrolysate contained 0.18 g/L HMF, 0.54 g/L formic acid, and 0.95 g/L acetic acid. The addition of NiO nanoparticles led to a progressive reduction in all inhibitors, with the most pronounced removal observed at the highest nanoparticle dosage. The HMF concentration declined steeply from 0.18 g/L to 0.04 g/L at 40 mg/100 mL NiO, corresponding to a 77.8% reduction. Formic acid removal followed a similar pattern, decreasing from 0.54 g/L to 0.10 g/L (81.5% reduction). Acetic acid, while less affected, still showed a notable decrease from 0.95 g/L to 0.46 g/L (51.6% reduction). This detoxifying effect could enhance microbial performance and support more efficient bioconversion in lignocellulosic bioethanol production systems. In parallel with current data, Bishop et al. (2025) showed that metallic nanoparticles reduced 5-HMF concentrations by 53.53% when acid-pretreated sugarcane molasses was used as a raw material. Moreover, in a previous study on NiO nanoparticle-assisted bioethanol production from potato peels, 5-HMF concentrations were significantly lower than in the control group (Sanusi et al. 2021). These results suggest that NiO nanoparticles may modulate inhibitor dynamics, possibly through adsorption, redox activity, or indirectly promoting microbial detoxification pathways (Dutta et al. 2023; Zhang and Li 2025). The significant reduction in HMF at moderate NiO concentrations highlights their potential as a functional additive to improve the fermentability of lignocellulosic hydrolysates. Overall, NiO nanoparticles demonstrate promise as a selective detoxification strategy for lignocellulosic hydrolysates, with potential to improve microbial fermentation performance. However, further studies should address the mechanism of inhibitor removal, evaluate possible nanoparticle leaching into the fermentation broth, and assess the impact of detoxification on ethanol yield and process economics.

**Table 1:** The effect of NiO on pretreatment (Pretreatment conditions: 1% H_2_SO_4_, 121 °C, 15 min, enzyme loading: 60 FPU/mL; fermentation time: 48 h for *K. marxianus*)

	NiO loading (mg/100 mL)	Reducing Sugar (g/L)	Inhibitor (g/L)		
			HMF	Formic acid	Acetic acid
Pumpkin pomace (150 g/L)	**0**	102.65^a^ ± 1.01	0.18^a^ ± 0.03	0.54^a^ ± 0.06	0.95^a^ ± 0.16
	**10**	125.74^b^ ± 0.62	0.16^a^ ± 0.02	0.38^b^ ± 0.07	0.88^a^ ± 0.17
	**20**	129.04^c^ ± 0.29	0.06^b^ ± 0.00	0.28^c^ ± 0.05	0.61^b^ ± 0.16
	**40**	124.26^b^ ± 0.33	0.04^b^ ± 0.00	0.10^d^ ± 0.00	0.46^b^ ± 0.08

*Different letters in the superscripts at the same column shows significant difference

In parallel with the decrease in inhibitor concentrations, supplementation with NiO nanoparticles significantly reduced sugar concentrations in PP hydrolysates. While the control without any NiO nanoparticles contained 102.65 g/L reducing sugars, supplementation with 10 and 20 mg/100 mL NiO increased concentrations to 125.74 and 129.04 g/L, respectively. At 40 mg/100 mL NiO, a slight decline to 124.26 g/L was observed, although values remained higher than the control. The decreasing trend in inhibitor concentrations with increased nanoparticle dosage indicates that NiO supplementation not only promotes sugar release but also contributes to a reduction in inhibitors in the hydrolysate.

Regarding their positive characteristics, integrating nanoparticles into fermentation systems has demonstrated significant improvements in both process efficiency and economic viability. For instance, iron-based nanoparticles, such as magnetite (Fe₃O₄), have been shown to enhance hydrogen yields by up to 53% during dark fermentation, primarily by facilitating electron transfer and improving microbial metabolism, thereby reducing the overall cost per unit of hydrogen produced (Pérez-Barragán et al. 2024). Similarly, the application of nanoparticles in syngas fermentation was reported to enhance gas–liquid mass transfer rates, which directly increases product yield while enabling reactor downsizing and energy savings—both of which contribute to a more cost-efficient process (Sajeev et al. 2023). Furthermore, techno-economic assessments of nanoparticle use in anaerobic digestion, especially with nickel and iron oxide nanoparticles, reveal substantial cost benefits (Jeyakumar and Vincent 2022). Similarly, in our current study, we showed that nanoparticle addition can reduce second-generation ethanol production costs. For example, in the current study, €7.51 was spent to produce 36.44 g/L of ethanol when 120 FPU/g of substrate enzyme was used. On the other hand, when the enzyme concentration was halved and 150 g/L PP medium was supplemented with 20 mg/100 mL NiO, only €3.88 was spent for both enzyme and nanoparticle. Using this approach, ethanol concentration increased by 17% to 42.64 g/L, demonstrating the beneficial effects of NiO nanoparticle addition. Therefore, it can be concluded that the use of NiO nanoparticles can offer a cost advantage of up to 49% (€3.68) per batch. Collectively, these findings demonstrate the potential of nanoparticles to improve the economic feasibility of various biofermentation processes by enhancing productivity, improving energy efficiency, and reducing operational costs.

In the current study, a reduction up to 49% in production cost was observed when the enzyme loading was decreased from 120 to 60 FPU/g. The literature indicates that acceptable saccharification can also be achieved with specific enzyme dosages, particularly when combined with improved pretreatment, lignin-blocking additives, or process strategies such as enzyme recycling and optimized mixing (Chen and Liu 2017). In this context, 60 FPU/g is often considered a practical threshold for achieving bioethanol production costs competitive with those of petroleum-derived fuels (Littlewood et al. 2013). Recent studies further show that specific additives or targeted pretreatment modifications can sustain high sugar yields, thereby reducing the enzyme-related cost contribution emphasized in techno-economic analyses (Hu et al. 2023). Given that enzyme cost remains a major barrier to commercial lignocellulosic ethanol production (Volynets et al. 2017), the improvement observed at 60 FPU/g compared with 120 FPU/g in this study should be interpreted as context-dependent rather than an absolute economic optimum. In addition, the results suggest that incorporating NiO nanoparticles beneficially influences both enzymatic hydrolysis and fermentation, indicating their potential as a cost-effective strategy to enhance bioethanol production.

### Comparison of ethanol production kinetics from pumpkin pomace by *K. marxianus*

The data illustrate how ethanol fermentation performance is influenced by biomass concentration, enzyme loading, and the addition of metal oxide nanoparticles (Table 2). Initially, increasing the biomass concentration from 50 to 150 g/L without enzymatic supplementation resulted in a marked decline in ethanol yield and productivity. For instance, the ethanol yield (Y_*P/S*_) dropped from 0.46 to 0.24 g/g, and the volumetric productivity (Q_*p*_) decreased from 0.75 to 0.18 g/L·h. This trend is likely attributable to the accumulation of inhibitory byproducts, mass transfer limitations, or increased medium viscosity at higher biomass loadings, which may hinder microbial activity Nguyen et al. 2017).

**Table 2:** All kinetic parameters belong to the *K. marxinanus* (Pretreatment conditions: 1% H_2_SO_4_, 121 °C, 15 min, **Medium 1**: only pretreated PP, **Medium-2**: enzymatically hydrolyzed PP without any supplementation, **Medium-3**: enzymatically hydrolyzed PP (60 FPU/g substrate) and 5 g/L peptone, 3 g/L yeast extract, 0.5 g/L MgSO₄·7 H₂O, 1 g/L KH₂PO₄, 0.1 g/L CaCl₂, and 0.05 g/L ZnSO₄ and **Medium-4**: enzymatically hydrolyzed PP (60 FPU/g substrate) and Nanoparticles)

Biomass loading (g/L)	Enzyme loading (FPU/mL)	Medium type	Nanoparticle loading (mg/100 mL)	Y_*P*/S max_ (g/g)	Q_*p* max_ (g/L.h)	Maximum theoretical ethanol yield (%)
50	**-**	**M1**	**-**	0.46^d^ ± 0.003	0.75^c^ ± 0.04	58.58^d^ ± 0.13
100	**-**	**M1**	**-**	0.45^d^ ± 0.04	0.72^c^ ± 0.01	61.63^d^ ± 0.27
150	**-**	**M1**	**-**	0.24^a^ ± 0.02	0.18^a^ ± 0.01	42.10^a^ ± 0.24
	**15**	**M2**	**-**	0.30^b^ ± 0.01	0.60^b^ ± 0.01	51.60^b^ ± 0.67
	**30**	**M2**	**-**	0.33^b^ ± 0.02	0.68^c^ ± 0.03	53.46^c^ ± 0.54
	**60**	**M2**	**-**	0.39^d^ ± 0.02	0.71^c^ ± 0.01	54.72^c^ ± 0.87
		**M3**	**-**	0.38^c^ ± 0.02	0.78^d^ ± 0.03	58.80^d^ ± 0.40
		**M4 (Fe**_**2**_ **O**_**3**_ **)**	**10**	0.36^c^ ± 0.01	0.64^b^ ± 0.03	54.51^c^ ± 0.23
			**20**	0.29^a^ ± 0.01	0.55^a^ ± 0.07	41.20^a^ ± 2.68
			**40**	0.18^a^ ± 0.00	0.33^a^ ± 0.03	26.46^a^ ± 0.19
		**M4 (NiO)**	**10**	0.37^c^ ± 0.02	0.75^c^ ± 0.04	59.03^c^ ± 2.05
			**20**	0.40^d^ ± 0.01	0.89^d^ ± 0.01	66.73^d^ ± 1.32
			**40**	0.19^a^ ± 0.03	0.41^a^ ± 0.01	30.61^a^ ± 0.21
		**M4 (ZnO)**	**10**	0.33^b^ ± 0.01	0.66^d^ ± 0.01	52.53^b^ ± 0.22
			**20**	0.31^b^ ± 0.002	0.64^b^ ± 0.03	48.53^b^ ± 0.42
			**40**	0.21^a^ ± 0.00	0.41^a^ ± 0.03	31.66^a^ ± 0.11
	**120**	**M2**	**-**	0.30^b^ ± 0.00	0.76^d^ ± 0.06	52.57^b^ ± 0.67

*Different letters in the superscripts at the same column shows significant difference

When enzyme loading was introduced at the highest biomass concentration, both yield and productivity improved. At 60 FPU/g substrate (Medium 2), the ethanol yield reached 0.39 g/g, and productivity approached 0.71 g/L.h, reflecting enhanced enzymatic hydrolysis and improved substrate accessibility. Furthermore, the productivity of Medium 3 was 0.78 g/L.h, with a theoretical ethanol yield of 58.8%, nearly matching values obtained at lower biomass levels without enzyme supplementation.

Supplementation with metal oxide nanoparticles under optimized conditions revealed a notable variation in performance depending on the type and concentration of nanoparticles used. NiO nanoparticles exhibited the most substantial positive impact. At 20 mg/100 mL NiO, the ethanol yield and productivity reached 0.40 g/g and 0.89 g/L.h, respectively, with a maximum theoretical yield of 66.73%. This suggests a potential role for NiO in mitigating fermentation inhibitors or enhancing microbial metabolism. In contrast, the same improving effect was not observed when Fe_2_O_3_ and ZnO nanoparticles were used. While moderate enhancements were observed at 20 mg/100 mL for both microbial growth, ethanol, and sugar production, higher concentrations (40 mg/100 mL) led to decreased yields, potentially due to nanoparticle toxicity or adverse interactions with microbial cells. Altogether, the findings highlight the intricate interplay among substrate loading, enzymatic activity, and nanoparticle supplementation for efficient bioethanol production.

## Conclusion

This study demonstrated the promising potential of utilizing various lignocellulosic biomass wastes—DFP and PP—as feedstocks for bioethanol production using yeast strains *S. cerevisiae*,* K. marxianus*, and *C. boidinii*. Among the substrates, PP yielded the highest ethanol with *K. marxianus*, especially at moderate biomass loadings, highlighting its suitability for sustainable biofuel production. Enzymatic hydrolysis efficiency and subsequent ethanol production were positively influenced by increasing cellulase concentrations up to an optimal level (60 FPU/g substrate), a value often cited as the critical limit for the economic viability of enzymatic hydrolysis of lignocellulose and bioethanol production. The incorporation of metal oxide nanoparticles, particularly nickel oxide (NiO), significantly enhanced bioethanol production by improving enzymatic hydrolysis and fermentation efficiency. Notably, this improvement was achieved even when the enzyme loading was halved from 120 FPU/g substrate to 60 FPU/g substrate. Ethanol titer increased from 36.69 g/L (Medium 3) to 42.64 g/L following the addition of 20 mg/100 mL NiO to the medium hydrolyzed with 60 FPU/g substrate. This concentration was therefore optimal for yield maximization, while higher levels (40 mg/100 mL) led to inhibition, likely resulting from nanoparticle toxicity. These findings indicate the critical importance of optimizing nanoparticle dosage to balance the biocatalytic benefits and potential microbial inhibition. Overall, the integration of nanoparticle-assisted hydrolysis and fermentation represents a viable, cost-effective strategy to increase bioethanol productivity from low-cost lignocellulosic wastes, advancing sustainable bioenergy technologies.

## Data Availability

No data was used for the research described in the article.
